# Angled Growth of the Dental Lamina Is Accompanied by Asymmetrical Expression of the WNT Pathway Receptor Frizzled 6

**DOI:** 10.3389/fphys.2017.00029

**Published:** 2017-01-31

**Authors:** Iveta Putnová, Hana Dosedělová, Vitezslav Bryja, Marie Landová, Marcela Buchtová, Jan Štembírek

**Affiliations:** ^1^Laboratory of Molecular Morphogenesis, Institute of Animal Physiology and Genetics, Academy of SciencesBrno, Czechia; ^2^Department of Anatomy, Histology and Embryology, University of Veterinary and Pharmaceutical SciencesBrno, Czechia; ^3^Department of Animal Physiology and Immunology, Institute of Experimental Biology, Masaryk UniversityBrno, Czechia; ^4^Department of Maxillofacial Surgery, University Hospital OstravaOstrava, Czechia

**Keywords:** FZD6, successional dental lamina, WNT signaling, planar cell polarity (PCP), odontoblast, ameloblast, osteoblast, epithelial remnants

## Abstract

Frizzled 6 (FZD6) belongs to a family of proteins that serve as receptors in the WNT signaling pathway. FZD6 plays an important role in the establishment of planar cell polarity in many embryonic processes such as convergent extension during gastrulation, neural tube closure, or hair patterning. Based on its role during hair development, we hypothesized that FZD6 may have similar expression pattern and function in the dental lamina, which is also a distinct epithelial protrusion growing characteristically angled into the mesenchyme. Diphyodont minipig was selected as a model species because its dentition closely resemble human ones with successional generation of teeth initiated from the dental lamina. We revealed asymmetrical expression of FZD6 in the dental lamina of early as well as late stages during its regression with stronger expression located on the labial side of the dental lamina. During lamina regression, FZD6-positive cells were found in its superficial part and the signal coincided with the upregulation of molecules involved in epithelial-mesenchymal transition and increased migratory potential of epithelial cells. FZD6-expression was also turned on during differentiation of cells producing hard tissues, in which mature odontoblasts, ameloblasts, or surrounding osteoblasts were FZD6-positive. On the other hand, the tip of successional lamina and its lingual part, in which progenitor cells are located, exhibited FZD6-negativity. In conclusion, asymmetrical expression of FZD6 correlates with the growth directionality and side-specific morphological differences in the dental lamina of diphyodont species. Based on observed expression pattern, we propose that the dental lamina is other epithelial tissue, where planar cell polarity signaling is involved during its asymmetrical growth.

## Introduction

Tooth development is a complex process that is dependent on reciprocal and strictly regulated interactions between the ectoderm-derived epithelium and cranial neural crest-derived mesenchyme (Thesleff et al., 1995). Numerous regulatory genes associated with all stages of tooth formation (patterning, morphogenesis, cytodifferentiation, and mineralization) belong to evolutionarily conserved signaling pathways. They are necessary for individual steps of odontogenesis and are regulated by a precise timing mechanism (Thesleff, 2003; Mitsiadis and Luder, 2011). The WNT signaling pathway has previously been demonstrated to play an important role in mouse as well as human tooth development (Sarkar and Sharpe, 1999; Sarkar et al., 2000; Handrigan and Richman, 2010). FZD members belongs to a family of proteins that serve as receptors in WNT signaling pathways (Fischer et al., 2007; Dijksterhuis et al., 2016; Wang et al., 2016). Their functions include activation of the FZD/β-catenin, FZD/Ca^+2^ and FZD/planar cell polarity signaling pathways (Schulte and Bryja, 2007). To date, 10 FZD proteins have been described in mammals (Schulte and Bryja, 2007; Dijksterhuis et al., 2016). While the expression of several ligands of WNT signaling during odontogenesis has been well-described (Cai et al., 2011; Lin et al., 2011; Wang et al., 2014), little attention has been paid to the expression of individual receptors. Here, we focus on FZD6, which is involved in PCP (planar cell polarity) signaling and is required for the transmission of polarity signals across the plasma membrane in epidermal cells (Wang et al., 2006).

Cellular communication mediated by WNT and FZD has been shown to be essential for proper embryonic development of invertebrates as well as vertebrates. FZD induction has been found in several developmental processes such as the polarized cell movements required for convergent extension during gastrulation in frog and fish (Borello et al., 1999), neural induction and patterning, cell proliferation, cell specification, stem cell differentiation, axonal outgrowth and guidance, and synaptogenesis (Logan and Nusse, 2004; Chien et al., 2009; Wang et al., 2010). Besides these early patterning processes, FZD3 and FZD6 have been reported redundantly to control neural tube closure and planar orientation of hair bundles on a subset of auditory and vestibular sensory cells. In the inner ear, these two proteins are located on the lateral faces of sensory and auxiliary cells in a pattern that correlates with the axis of planar polarity (Wang et al., 2006). The polarity of FZD6 localization with respect to the asymmetric position of the kinocilium is reversed between vestibular hair cells in the cristae of the semicircular canals and auditory hair cells in the organ of Corti (Wang et al., 2006). FZD6 controls macroscopic hair patterning in the mouse and is also expressed in the skin and hair follicles (Guo et al., 2004; Wang et al., 2010; Chang et al., 2016).

Mutations in PCP genes lead to a wide range of developmental defects, including a shortened body axis, a widened neural plate, and neural tube defects (NTDs; Simons and Mlodzik, 2008). In the case of targeted deletion of the *Fzd6* gene, stereotyped whorls on the hind feet, variable whorls and tufts on the head and disorientation of hairs on the torso are evident (Guo et al., 2004). In the *Fzd6*^−/−^ mouse, the orientations of the earliest born hair follicles are uncorrelated, but over time the follicles reorient to create patterns, which are typical for different body regions and are characterized by a high degree of local arrangement (Wang et al., 2010). Fifty percent of male newborns, but not female *Fzd6*^−/−^ mice, displayed abnormal claw morphology. The claws are easily lost with age or under increased mechanical stress. The claw disappears or become rudimentary on the hind limbs at the age of 2–3 months. The reason for the significant misbalance between sexes is unknown but it could be due to the more aggressive behavior of males (Fröjmark et al., 2011). Similarly in humans, loss-of-function mutations caused recessive nail dysplasia (Fröjmark et al., 2011; Naz et al., 2012). While several ectodermal derivates have been found to be affected in transgenic mouse lines or humans with defective FZD6, to date, no tooth phenotype has been described.

Here, we analyzed the expression of FZD6, a transmembrane protein of the WNT family, that is known to regulate the number of epithelial differentiation-related genes. During human odontogenesis, *Fzd6* was shown to exhibit weak mRNA expression in the dental epithelium of incisors and molars at 8 and 12 weeks of gestation (Wang et al., 2014). Later during 15 week, *Fzd6* was observed in the inner and outer enamel epithelium and in the surrounding mesenchymal cells (Wang et al., 2014). However, the distribution of FZD6 on protein level has not been analyzed yet. We focused on premolar development in diphyodont dentition during early as well as late mineralization stages of odontogenesis to determine the distribution of its expression throughout development. Labio-lingual differences during the initiation and regression of dental lamina were analyzed to uncover signaling involved in asymmetrical morphology and growth of the lamina. Therefore, the main aim of our study was to describe the expression pattern of FZD6 at the protein level during early odontogenesis in the minipig dentition with a special focus on the asymmetric distribution of FZD6 in the dental lamina during its angled growth and regression. Furthermore, changes in FZD6-positivity in odontoblasts and ameloblasts during their differentiation were determined.

## Materials and methods

### Embryonic material

Selected developmental stages of the minipig (E29, E30, E36, E56, E67) were used to analyse the expression of FZD6 during odontogenesis. Minipig embryos and fetuses were obtained from Liběchov animal facility (Liběchov, Czech Republic). The day after insemination was established as day 1 of gestation. Staged embryos and fetuses were obtained by hysterectomy. All samples were fixed in 4% neutral formaldehyde and decalcified in 12.5% EDTA in 4% PFA until the mandibular bones of embryos were soft enough for further processing. Sections were stained with Haematoxylin-Eosin and alternative slides were used for immunohistochemical labeling. All procedures were conducted following a protocol approved by the Laboratory Animal Science Committee of the Institute of Animal Physiology and Genetics, Academy of Sciences (approval no. 020/2010, Liběchov, Czech Republic).

### Immunohistochemical analysis

For detection of FZD6-positivity, we performed immunohistochemical labeling. After deparaffinization and rehydration, antigen retrieval was performed in a water bath (97°C) in citrate buffer (pH = 6) for 20 min. Blocking serum was applied to the sections for 20 min and slides were incubated for 1 h at room temperature with primary FZD6 antibody (cat. no. G260, Antibodies online, 1:200 dilution). The secondary antibody was applied for 30 min. Streptavidin-FITC complex (1:250 dilution, cat. no. 554060, BD Pharmigen, Franklin Lakes, USA) was used for visualization of FZD6-positive cells (30 min). DAPI (cat. no. P36935, Invitrogen, Oregon, USA) or DRAQ5 (1:500 dilution, cat. no. 62254, Thermo Scientific, USA) were applied for the counterstaining. The photos taken under a fluorescence microscope Leica DM LB2 (Leica Microsystems, Germany) were merged together in Adobe Photoshop 7.0 (USA). High power images were taken on confocal microscope Leica SP5 using 40x (air) objectives (Leica Microsystems, Germany) with Leica Application Suite software.

## Results and discussion

### Asymmetrical expression of FZD6 at early stages of odontogenesis

**Figure 1:**
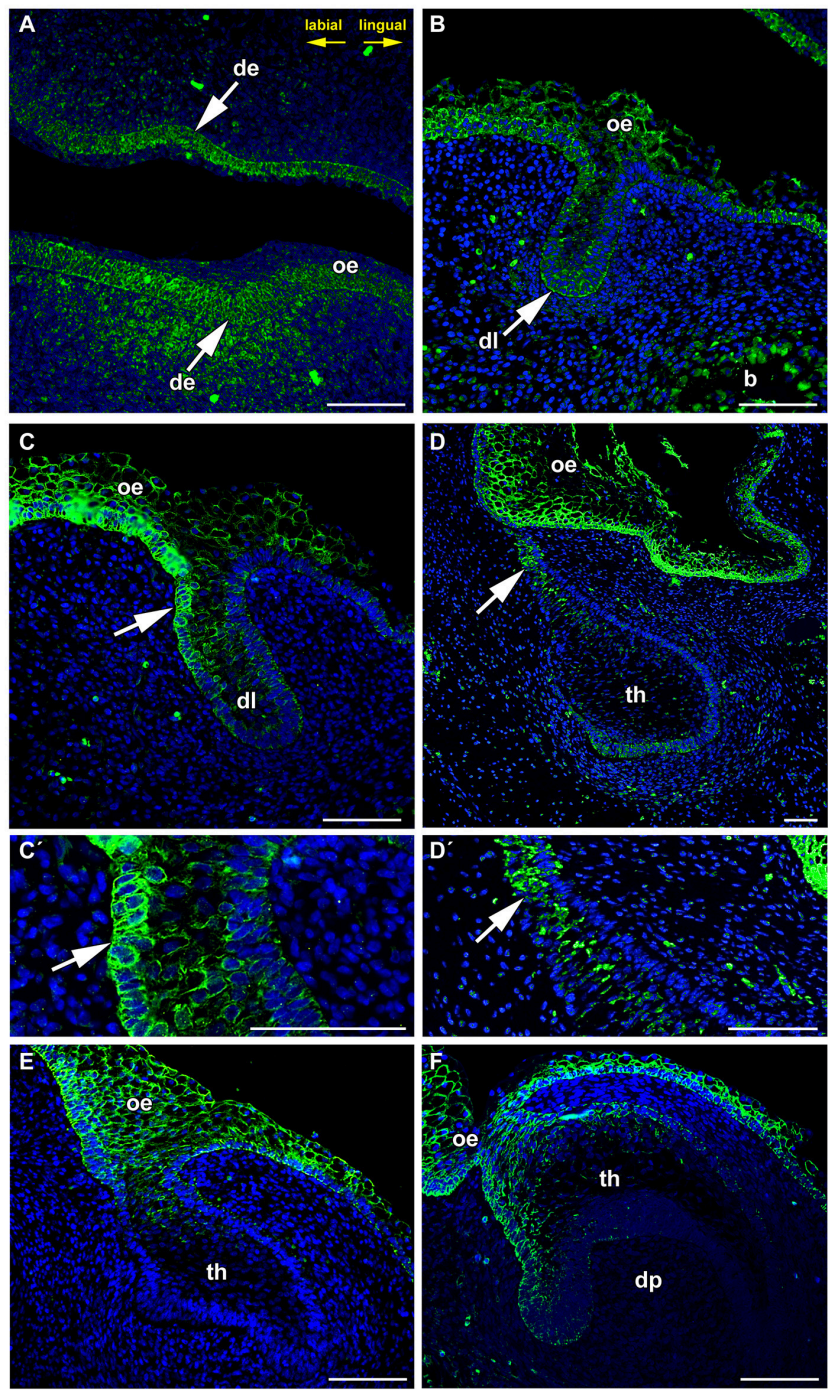
**FZD6 expression is asymmetrical in the dental lamina. (A)** At epithelial thickening stage, the oral epithelium and surrounding mesenchyme are FZD6-positive. (**B,C,C**′ in detail) Later, FZD6-positive signal is located on the labial side (arrow) of the protruding dental lamina. (**D,D**′ in detail) At dental bud stage, stronger FZD6-immunopositivity (arrow) was detected on the labial side of dental germ. **(E,F)** At bell stage, the labial cervical loop is more positive that the lingual one. Almost no signal was visible in the stellate reticulum. The dental papilla and surrounding mesenchyme were FZD6-negative. b, bone; de, dental epithelium; dl, dental lamina; dp, dental papilla; oe, oral epithelium; th, tooth. Scale bar = 100 μm.

PCP components are often localized asymmetrically in different types of cells. The Frizzled family was found to be required for producing the correct orientation of cuticular bristles and hairs (Guo et al., 2004). This process is referred to as tissue or planar polarity (Gubb and García-Bellido, 1982; Vinson et al., 1989). Such polarization is often precisely coordinated relative to the axes of a tissue or organ, but the mechanisms underlying this regulation are still poorly understood. As exact coordination of tissue polarity in the labio-lingual axis is necessary to direct the angled growth of dental lamina into the mesenchyme, we wondered whether protein expression FZD6 can be associated with morphological side-related differences in the dental lamina. Indeed, we observed significant differences in the level of FZD6-positivity in distinct areas of the dental lamina at all stages of minipig odontogenesis. Already at the epithelial thickening stage, a strong FZD6 signal was apparent on the labial side of the oral epithelium and in the dental epithelium, especially in its basal layer (Figure 1A). The signal became weaker toward its lingual side. Distinct expression was also obvious in the mesenchyme surrounding the epithelial thickening, where more FZD6-positivity was noticeable in the labial area (Figure 1A). Later, during dental lamina growth into the mesenchyme, stronger expression was observed also on the labial side of the oral dental interface in comparison with the lingual area (Figures 1B,C,C′). At the dental bud stage, FZD6 was only weakly expressed inside the tooth anlagen (Figures 1D,D′) while FZD6-immunopositivity was detected in the dental lamina connecting the tooth to the oral epithelium, where stronger expression was located on the labial side (Figures 1D,D′).

At the cap stage (Figure 1E), distinct expression was found throughout all thicknesses of the oral epithelium and in the dental lamina, but was not apparent inside the tooth anlagen. The signal spread into the cervical loop area during the early bell stage (Figure 1F). Almost no signal was visible in the stellate reticulum (Figure 1F). The dental papilla and surrounding mesenchyme were FZD6-negative. FZD6-signal was abundant in the basal layer of the oral epithelium at all analyzed stages and became localized to the superficial area of membrane in more differentiated superficial layers of the epithelium (Figure 1).

It is known that FZD6 is transducing signals in non-canonical WNT pathway and Wnt5a is one of its representative, which signals upstream of PCP pathway in mammals (Moon et al., 1993; Kilian et al., 2003). It was shown that WNT5a regulates tooth growth, cusp patterning and odontoblast differentiation in developing mouse molars and incisors (Lin et al., 2011). Previously, WNT5a expression was found not only in the dental mesenchyme but also in the dental epithelium in mouse during E14–E17 (Cai et al., 2011). However, we detected FZD6 even at very early stages of epithelial thickening and in the surrounding mesenchyme, and therefore another WNT ligand probably activates signaling at these early stages, which will be necessary to further analyse in future.

FZD can also mediate canonical signaling with activation of β-catenin. WNT3a is strongly expressed in the inner enamel epithelium of humans during the bell stage (Wang et al., 2014). In mice, *Wnt3a* is expressed in the enamel knot at the cap stage (Millar et al., 2003). These expression patterns do not seem to correlate with our observations of a strong signal located in the superficial part of the tooth anlagen whereas deeper parts including enamel knots and cervical loops were FZD6-negative (Figure 1).

FZD6 was also distributed in distinct areas of the mesenchyme surrounding epithelial thickenings during the early stages of odontogenesis; however, mesenchymal expression was downregulated and only small spots were visible on the mesenchymal cell surface at older stages. On the other hand, FZD6 was mostly expressed in the epithelium with uniform expression throughout the whole thickness of the oral epithelium. This finding is consistent with observations in FZD6 knockout mice, where numerous genes encoding keratins, keratin-associated proteins and transglutaminases and their substrates were significantly downregulated (Cui et al., 2013). Therefore, the mesenchymal expression pattern is dissimilar to the epithelial signal indicating distinct role of FZD signaling in the epithelium in contrast to the mesenchyme.

### FZD6 expression in the successional dental lamina

**Figure 2:**
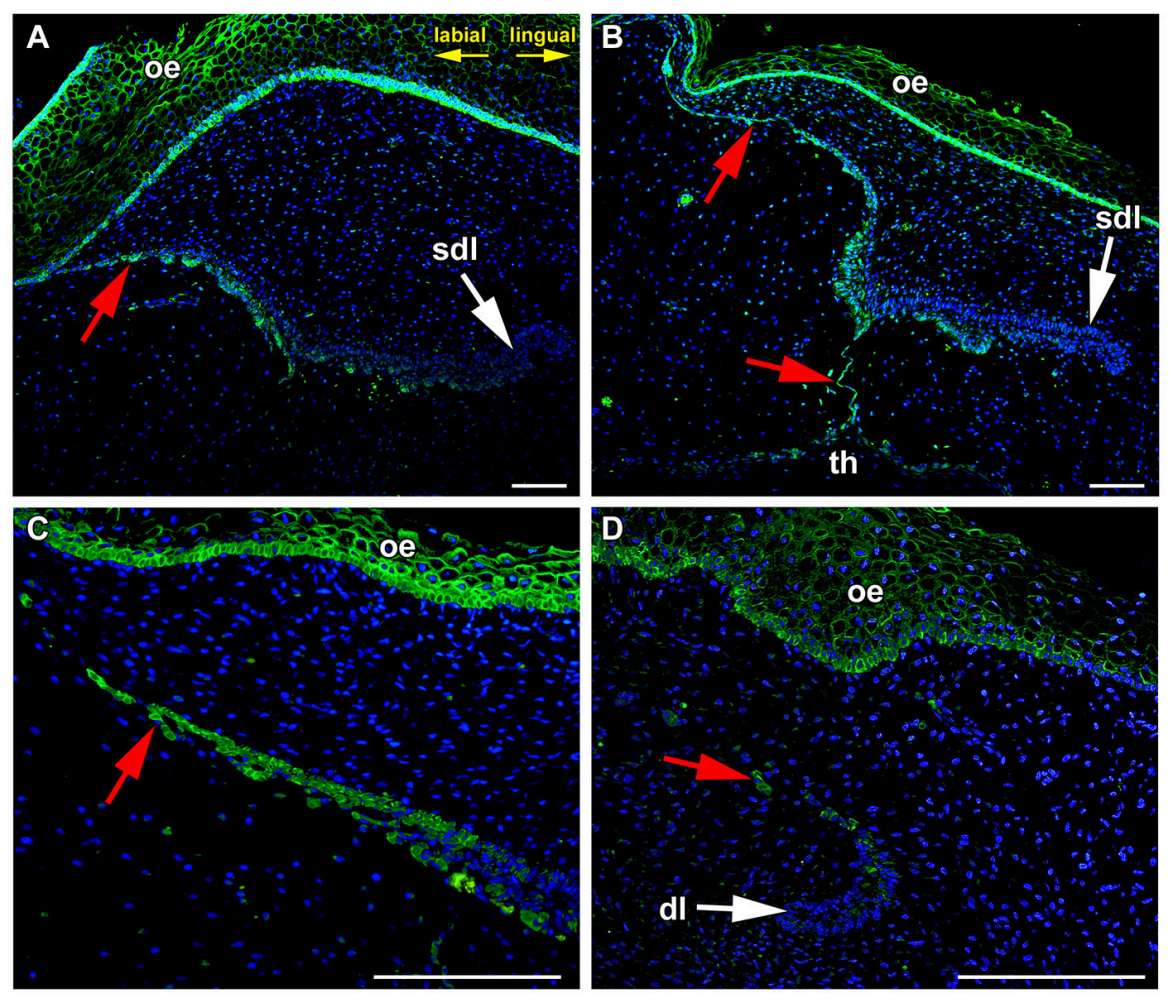
**The tip of growing successional dental lamina is FZD6-negative. (A)** In the most superficial area, flat cells connecting the lamina to the oral epithelium were FZD6-positive. **(B)** Cells on the labial side separating from the lamina were also FZD-positive similarly to the stalk of very flat cells connecting the dental lamina to the tooth. However, the apical tip of the successional lamina was FZD6-negative. **(C)** When dental lamina disconnected from the oral epithelium, the stronger positivity of Fzd6 was evident in the superficial fragments and epithelial clusters of cells. **(D)** Rudimental interdental lamina exhibited only weak FZD6 expression. dl, dental lamina; oe, oral epithelium; sdl, successional dental lamina; th, tooth; red arrow, epithelial remnants. Scale bar = 100 μm.

**Figure 3:**
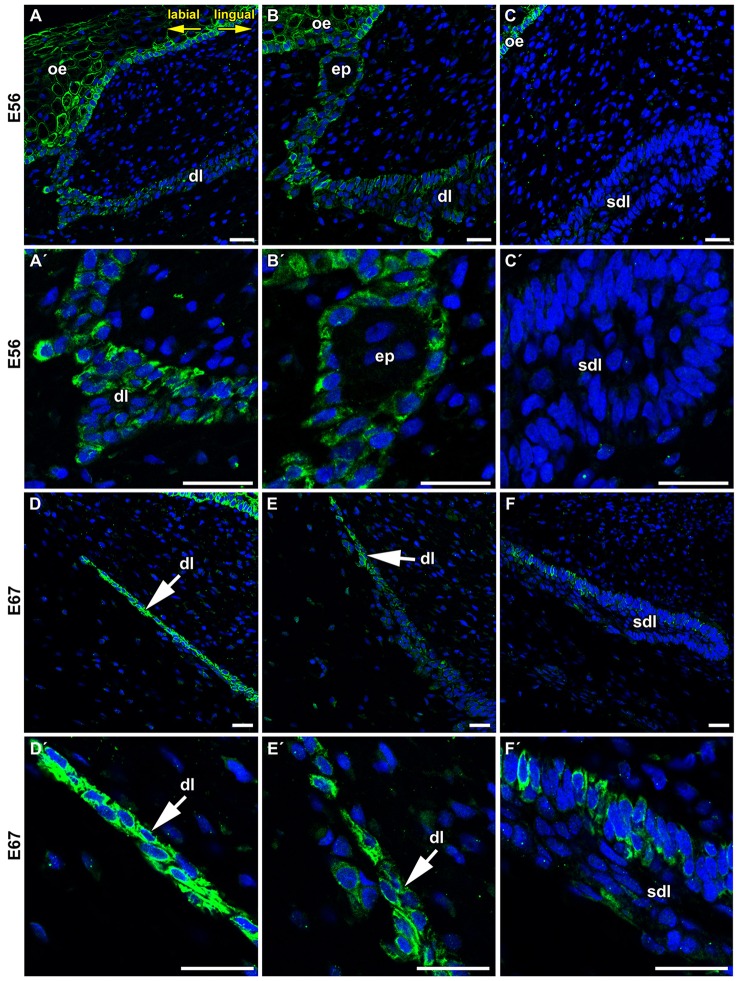
**Details of FZD6 expression in the dental lamina. (A,A′,B,B′)** Stronger expression of FZD6 was observed in the superficial area of the dental lamina, which was still connected to the oral epithelium. **(C,C**′**)** No FZD6 expression was found in deeper successional dental lamina while spotted pattern of expression was observed in surrounding mesenchymal cells. **(D,D**′**)** Later in development, superficial part was disconnected from the oral epithelium and cells exhibited strong FZD6-positivity. **(E,E**′**)** Expression was gradually lost in the central part of the lamina. **(F,F**′**)** The tip of the successional lamina was negative similar to younger stage, only few positive cells were found in more superficial position. dl, dental lamina; ep, epithelial pearl; oe, oral epitheliuml; sdl, successional dental lamina. Scale bar = 25 μm.

During later stages of odontogenesis, the dental lamina protruded deeply into the mesenchyme and morphological changes were obvious in its superficial part (Figure 2). In the most superficial area, cells connecting the lamina to the oral epithelium were FZD6-positive (Figures 2A,B, 3A,A′,B,B′). Expression in superficial cells even increased following disconnection of the lamina from the oral epithelium (Figures 2C, 3D,D′,E,E′). Cells on the labial side separating from the lamina were strongly FZD6-positive similar to the stalk of very flat cells connecting the dental lamina to the tooth (Figures 2A–C). On the other hand, the apical tip of the successional lamina was FZD6-negative (Figures 2A–D, 3C,C′,F,F′). Therefore, there are significant differences in FZD6 expression through the dental lamina with higher expression maintained in its superficial layers.

While, the most distal tip of the successional dental lamina was FZD6-negative, β-catenin was previously described in the tip and lingual side of the dental lamina in snake and alligator (Wu et al., 2013) and transcription factor Lef1 (Wnt/β-catenin pathway target gene) in corn snake and python dental lamina (Handrigan and Richman, 2010; Gaete and Tucker, 2013). WNT ligands that signal through β-catenin are involved in stem/progenitor self-renewal and maintenance of cells in a proliferative and undifferentiated state while non-canonical signaling promotes their differentiation (Liu et al., 2009; Grumolato et al., 2010). As non-canonical signaling is known to inhibit WNT/β-catenin canonical signaling (Topol et al., 2003; Mikels and Nusse, 2006), this asymmetrical expression of non-canonical and canonical WNT molecules and the balance among them is critical for the regulation of dental progenitor cell lines similar to that shown in other systems (Grigoryan et al., 2008). Based on our evidence, FZD6 is not expressed in the areas of dental lamina with high cell proliferation or proposed localization of progenitor cells. Therefore, canonical and non-canonical WNT signaling exhibits distinct asymmetrical expression pattern through the lamina, which seems to be aligned with side-specific differences during successional dental lamina formation.

### FZD6 was strongly expressed in the epithelial remnants and pearls during dental lamina regression

The minipig similar to human has a diphyodont type of dentition, where only two generation of teeth are initiated. During embryonic period, the dental lamina undergoes major morphological changes and becomes thinner and disconnected from the oral epithelium. Cells are elongated in the superficial area of the dental lamina (Buchtová et al., 2012). Furthermore, they are elongated in the area of the dental stalk connecting the tooth anlagen to the dental lamina. After the dental lamina had disconnected from the oral epithelium, stronger expression of FZD6 was evident in the superficial epithelial cells. In deeper parts of the lamina, the expression of FZD6 was apparent on the side facing the tooth, which undergoes regression (Figure 2C). There were also differences in the level of expression along the jaw axis. In the area between teeth, rudimental interdental lamina exhibited only weak FZD6 expression located in the most superficial area (Figure 2D).

**Figure 4:**
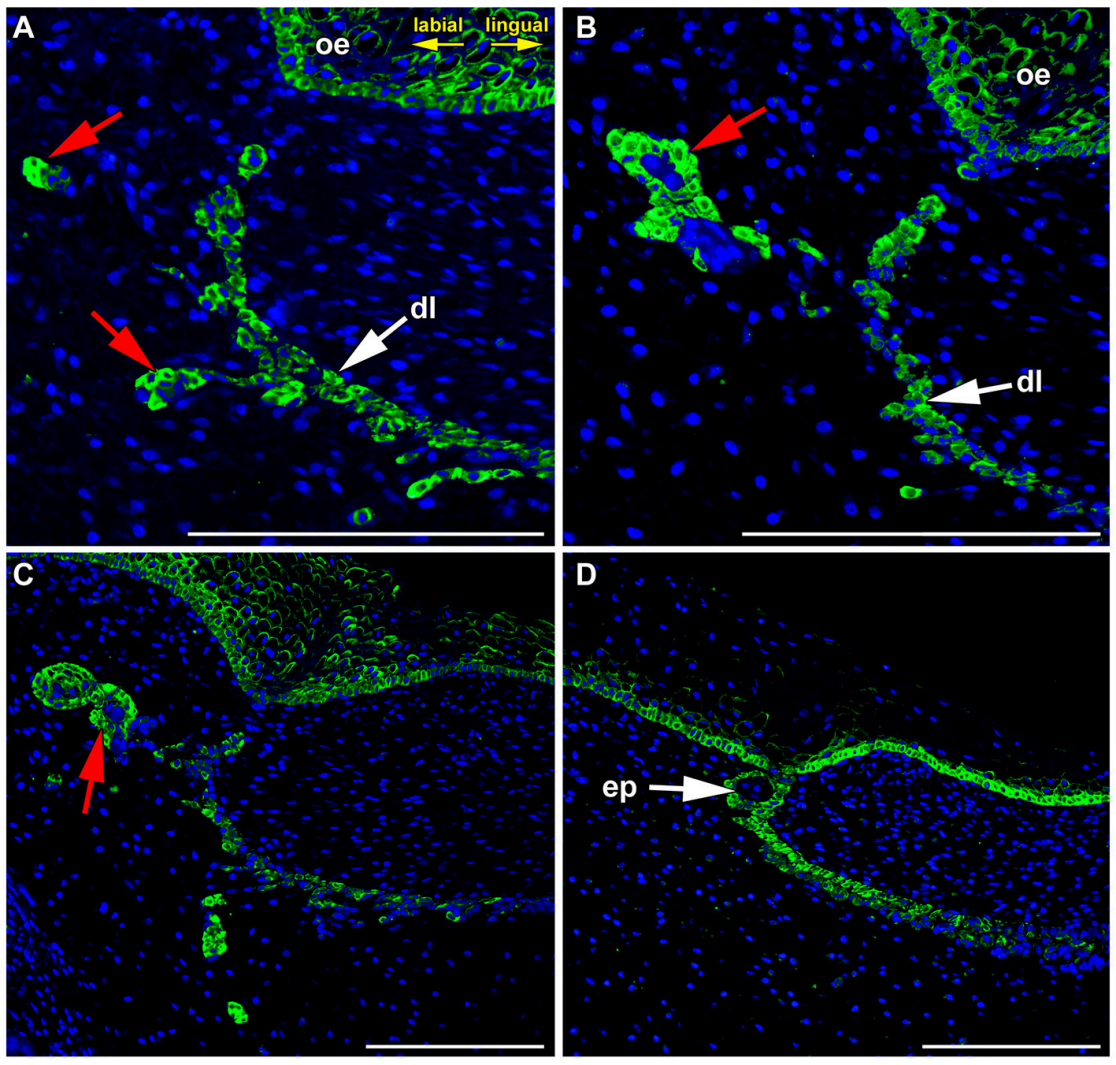
**FZD6 expression in epithelial remnants and pearls. (A,B)** Localized and cytoplasmatic expression of FZD6 was found in epithelial remnants. **(C)** The expression was found in the epithelial clusters during process of pearl formation as well as in the basal layer of already formed pearls **(D)**, while their central area was negative. ep, epithelial pearl; red arrow, epithelial remnants. Scale bar = 100 μm.

Later during dental lamina regression, the dental lamina become fragmented, and only occasional epithelial islands remain in the superficial area (Figure 4A). Some of these fragments undergo further morphological changes and epithelial pearls become visible along the jaw (Buchtová et al., 2012). Expression was also found in the epithelial clusters during the process of pearl formation (Figure 4C) as well as in the basal layer of already formed pearls (Figure 4B), while the central area was negative (Figure 4D).

Interestingly, distinct expression was obvious especially in the basal layers of the lamina epithelium facing the tooth where clusters of elongated cells were moving out of the dental lamina. The expression of FZD6 during lamina regression coincides with the upregulation of molecules involved in epithelial-mesenchymal transition of epithelial cells (Buchtová et al., 2012). Our observations that cells moving from the dental lamina are FZD6-positive is consistent with the previously known role of PCP proteins in notochord or somite cell elongation during their convergent extension (Keller et al., 2000; Keller, 2002; Seifert and Mlodzik, 2007) as well as regulation of cell polarity and directed motility during gastrulation in frogs and fish, and neural tube and eyelid closure in mammals (Wang et al., 2006; Seifert and Mlodzik, 2007).

### Frizzled 6 in differentiation of hard-tissue producing cells

Both canonical and non-canonical WNT pathways were previously shown to be involved in the differentiation of hard-tissue producing cells (Millar et al., 2003; Lin et al., 2011; Sakisaka et al., 2015). WNT signaling was shown to promote the differentiation of dental follicle cells into the cementoblast or osteoblast phenotype (Peng et al., 2010; Du et al., 2012; Sakisaka et al., 2015; Nemoto et al., 2016). Moreover, Wnt5a overexpression promotes the differentiation of dental papilla cells and increased the expression of mineralization-related genes (Peng et al., 2010).

**Figure 5:**
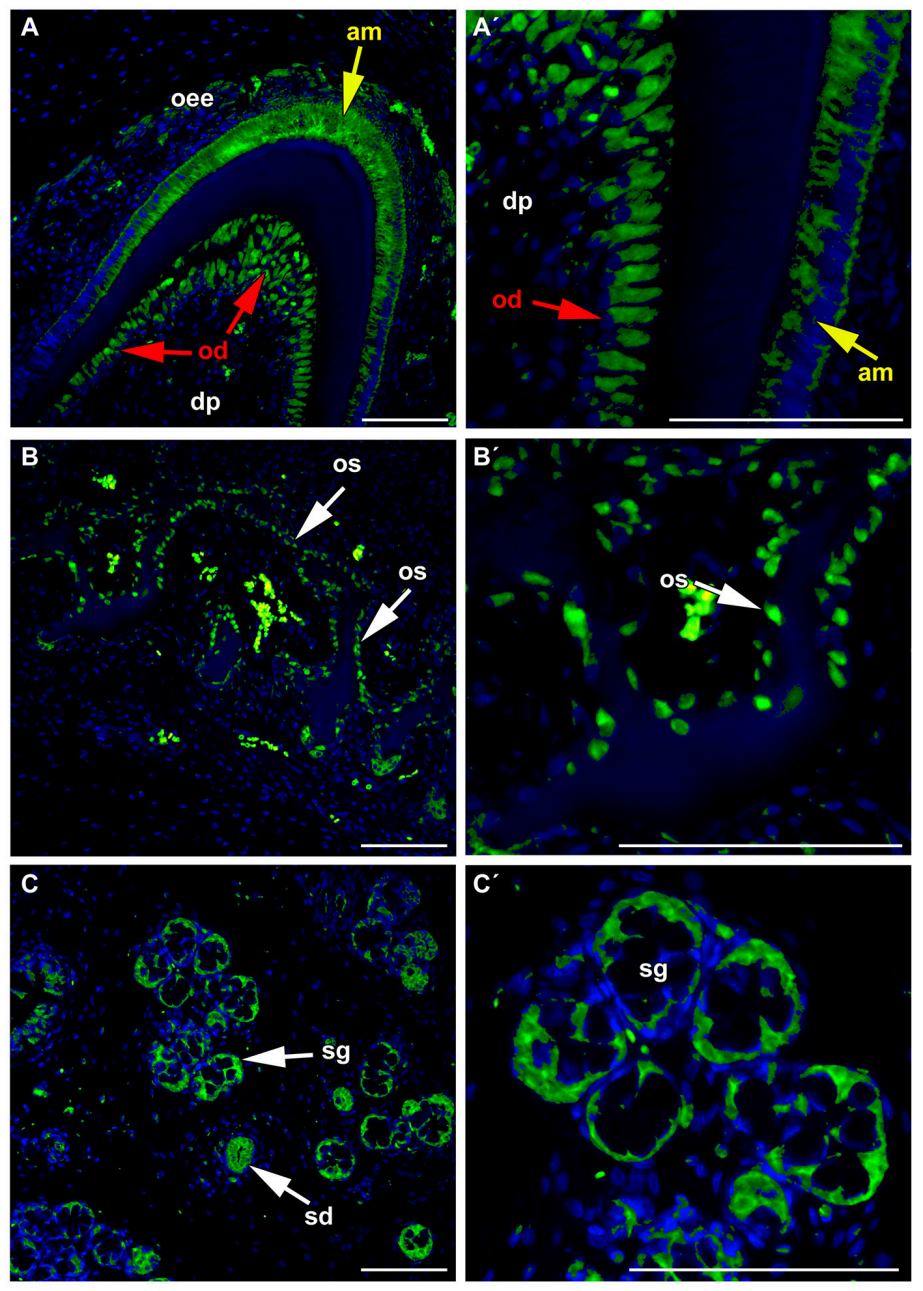
**FZD6 in differentiated ameloblasts and odontoblasts. (A,A′)** Differentiated and secretory ameloblasts were FZD6-positive while non-differentiated cells of the inner enamel epithelium were negative. Similar pattern was observed in the dental papilla where differentiated odontoblasts exhibiting dentin production were FZD6-positive. FZD6 signal was localized at different stages of ameloblasts differentiation after their elongation. **(B,B**′**)** FZD6 signal was located also in the structures surrounding teeth such as alveolar bone where osteoblasts were positive and secretory cells of salivary gland or their ducts **(C,C**′**)**. am, ameloblast; dp, dental papilla; od, odontoblast; oee, outer enamel epithelium; sg, salivary gland. Scale bar = 100 μm.

In agreement with these findings, FZD6 expression was switched on during the differentiation of odontoblasts, ameloblasts, and osteoblasts in minipigs (Figure 5) at time when the production of hard tissues started. Differentiated and secretory ameloblasts were FZD6-positive while non-differentiated cells of the inner enamel epithelium were negative (Figures 5A,A′). A similar pattern was observed in the dental papilla where differentiated odontoblasts exhibiting dentin production were FZD6-positive (Figures 5A,A′). Almost no signal was found in the stellate reticulum or stratum intermedium (Figure 5A). On the other hand, the outer enamel epithelium was positive, especially the superficial clusters of cells during disruption of the enamel organ by blood vessels. FZD6 signal was also located in the structures surrounding teeth in the jaw such as alveolar bones, in which osteoblasts were positive (Figures 5B,B′) along with the secretory area of salivary glands or their ducts (Figures 5C,C′).

While the tooth phenotype of FZD6 mutant mice has not yet been described, *Wnt5a*-deficient mice exhibit delayed odontoblast differentiation (Lin et al., 2011). Abnormal morphology of ameloblasts and defective odontoblast differentiation with absence of predentin formation were also found in *Ror2* mutant mice (Lin et al., 2011) in which ROR2 can serve as an alternative WNT receptor (Oishi et al., 2003). Odontoblasts in *Wnt5a*-deficient mice were shorter and thicker than in control animals. Similarly in *Ror2*-deficient mice, odontoblasts were polarized but appeared to be shorter in comparison to littermate control animals (Lin et al., 2011). While *Wnt5a* is expressed in the dental mesenchyme, its receptor is also expressed in the epithelium. It was proposed that another receptor must be involved in WNT5a-mediated signaling as defects in tooth development in *Ror2* mutants occur much later than observed in WNT5a-deficient mice (Lin et al., 2011). Based on our observations, FZD6 could be one of these receptors.

Odontoblasts and ameloblasts are highly polarized cells with a characteristic morphology and arrangement of cellular compartments. In odontoblasts, FZD6 was expressed asymmetrically only on the side facing the dentin (Figures 5A,A′). In ameloblasts, FZD6 was observed on both sides of differentiated cells, and later, the signal showed a more uniform distribution through the cells. Differentiation of ameloblasts and odontoblasts is characterized by elongation of the cells and establishment of their polarity. Actin filament bundles exhibit a polarized distribution in rat ameloblasts and are abundant at the ameloblast junction area (Nishikawa and Kitamura, 1985, 1986). As actin cytoskeleton organization is downstream of the PCP signaling pathway, it is possible that FZD6 can be involved in the rearrangement of cellular polarity in the odontoblasts and ameloblasts during their differentiation.

## Conclusions

**Figure 6:**
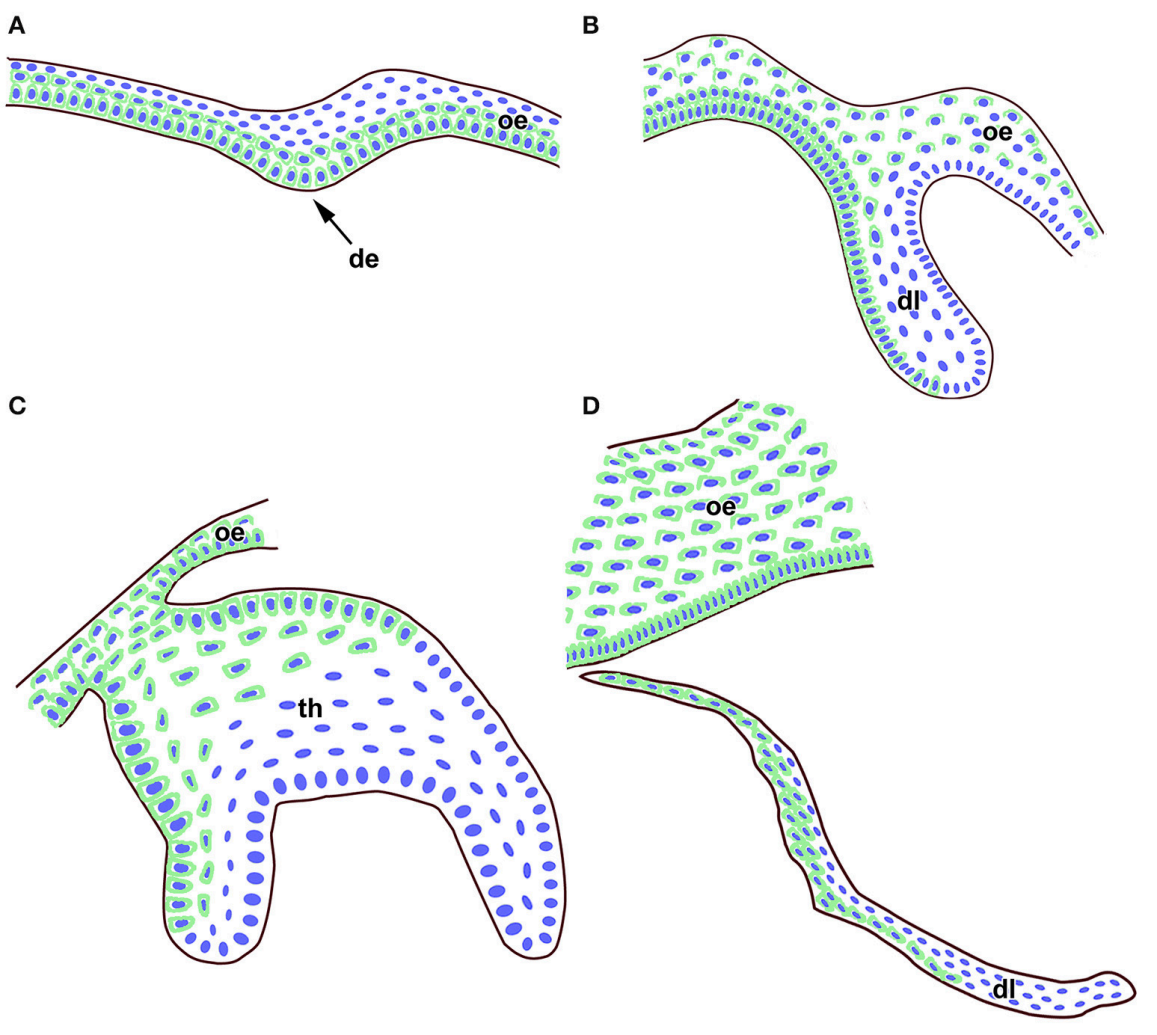
**Schema of FZD6 protein expression through odontogenesis. (A)** At the epithelial thickening stage, a strong FZD6 signal was apparent especially in basal layers of the oral and future dental epithelium. **(B)** Later, FZD6-positivity was detected in the dental lamina with stronger expression on the labial side. **(C)** At the bell stage, distinct expression was found throughout the oral epithelium and in the labial cervical loop area. Almost no signal was visible in the stellate reticulum. **(D)** The tip of successional dental lamina was FZD6-negative while cells in the superficial part of the lamina were FZD6-positive. Nuclei are labeled by blue and FZD6 expression in green through the epithelial tissues. de, dental epithelium; dl, dental lamina; oe, oral epithelium; th, tooth.

In summary, FZD6 was expressed asymmetrically in the dental lamina at early as well as late stages of diphyodont dentition (Figure 6). We observed significant differences in the level of FZD6 expression in distinct areas of the dental lamina with stronger expression in the labial and superficial parts (Figure 6). The apical tip of the successional lamina was FZD6-negative. During dental lamina regression, labial cells separating from the lamina were strongly FZD6-positive similar to the stalk of very flat cells connecting the dental lamina to the tooth germ. On the other hand, FZD6 was not expressed in the areas of dental lamina with high cell proliferation or proposed localization of progenitor cells.

FZD6 expression was also switched on during the differentiation of odontoblasts, ameloblasts, and osteoblasts in minipigs at time when the production of hard tissues started. In odontoblasts, FZD6 was expressed asymmetrically only on the side facing the dentin therefore it is possible that FZD6 can be involved in the establishment of cellular polarity in the odontoblasts during their differentiation. However, the exact role of FZD6 in the growth directionality of the lamina and differentiation of hard tissue producing cells has to be proven experimentally in future.

## Author contributions

MB and VB designed the project. IP, HD, ML, and JS performed and interpreted experiments. IP, MB, and JS wrote manuscript with contribution of VB. All authors approved the manuscript.

### Conflict of interest statement

The authors declare that the research was conducted in the absence of any commercial or financial relationships that could be construed as a potential conflict of interest.
